# SUBJECTIVE VIEWS OF AGING AND SOCIAL RELATIONSHIPS AMONG OLDER ADULTS: THEORY OF MIND AS A MEDIATOR

**DOI:** 10.1093/geroni/igac059.898

**Published:** 2022-12-20

**Authors:** Yoav Bergman, Amit Shrira, Orel Swisa

**Affiliations:** Ashkelon Academic College, Ashkelon, HaDarom, Israel; Bar-Ilan University, Ramat Gan, HaMerkaz, Israel; Bar-Ilan University, Ramat-Gan, HaMerkaz, Israel

## Abstract

Research has demonstrated the importance of subjective views of aging (VoA) for older adults’ well-being. However, little is known regarding how understanding others’ emotional states may affect this association. Accordingly, we examined whether Theory of Mind (ToM), or the ability to comprehend others’ emotions, may mediate the effect of VoA on older adults’ social functioning. Thirty community-dwelling older adults (mean age= 71.23, SD= 8.57, 50% women) filled out daily questionnaires concerning ageist attitudes, attitudes toward aging, and satisfaction with social relationships for two weeks. Positive VoA (i.e., low ageist attitudes and positive attitudes toward aging) were associated with increased ToM and higher satisfaction with social relationships. Moreover, ToM was a significant mediator for the VoA-social relationships link, as positive VoA on a given day were indicative of higher ToM levels, which were associated with increased positive social relationships. Results are discussed from the perspective of Socioemotional Selectivity Theory.

